# Anesthetic Considerations in Off-Pump Coronary Artery Bypass Grafting: A Narrative Review

**DOI:** 10.3390/jcm15218499

**Published:** 2026-11-01

**Authors:** Gentian Huti, Artan Jahollari, Asead Abdyli, Rudin Domi, Driola Hoxha, Besnik Filaj, Oliatina Demiri, Ervisa Abdyli, Daniele Biasucci, Filadelfo Coniglione, Fabio Guarracino, Vojislava Neskovic, Idit Matot, Krenar Lilaj, Vedat Eljezi, Anne-Marie Camilleri-Podesta

**Affiliations:** 1 Department of Anesthesia and Intensive Care Medicine, American Hospital 3, Tirana 1005, Albania; 2 Department of Cardiovascular Surgery, American Hospital 3, Tirana 1005, Albania; artan.jahollari@spitaliamerikan.com; 3 Department of Surgery, University of Medicine, Tirana 1005, Albania; 4 Department of Clinical Science and Translational Medicine, ‘Tor Vergata’ University of Rome, 00133 Rome, Italy; danielebiasucci@gmail.com (D.B.);; 5 Department of Medical, Dental and Biotechnological Sciences, University Gabriele d’Annunzio Chieti-Pescara, Anesthesia, Resuscitation and Intensive Care, University Hospital of Chieti, 66100 Chieti, Italy; 6 Clinic for Anesthesia and Critical Care, Military Medical Academy, Faculty of Medicine MMA, 11040 Belgrade, Serbia; vojkan43@gmail.com; 7 Division of Anesthesiology, Intensive Care and Pain, Tel Aviv Medical Center, Faculty of Medicine, Tel Aviv University, Tel Aviv 6423906, Israel; 8 Department of Perioperative Medicine, CHU Gabriel-Montpied, 63003 Clermont-Ferrand, France; 9 Department of Anesthesia and Intensive Care, Mater Dei Hospital, MSD 2090 Msida, Malta

**Keywords:** coronary artery bypass, coronary artery bypass, off-pump, hemodynamic monitoring, echocardiography, transesophageal, analgesia, thoracic epidural

## Abstract

**Background**: Off-pump coronary artery bypass grafting (OPCAB) avoids cardiopulmonary bypass (CPB) and may reduce selected perioperative complications, but its technical and hemodynamic demands remain important limitations. **Methods**: We narratively reviewed the literature indexed in PubMed/MEDLINE and Scopus, supplemented by Google Scholar and reference-list screening, covering January 2000 to August 2026. Priority was given to systematic reviews, meta-analyses, randomized trials, prospective studies, guidelines, and relevant landmark reports. **Results**: OPCAB may be particularly useful in selected patients at increased risk from CPB or aortic manipulation. Cardiac displacement and coronary stabilization can cause rapid changes in preload, ventricular function, myocardial perfusion, and cardiac output, making invasive monitoring, transesophageal echocardiography, and coordinated hemodynamic management central to anesthetic care. Current evidence does not establish the superiority of volatile anesthesia or total intravenous anesthesia. Comparative studies suggest potential reductions in perioperative stroke, transfusion, renal complications, and hospital stay, although potential stroke benefits depend on the aortic-handling strategy and should not be attributed to avoidance of CPB alone. Concerns remain regarding completeness of revascularization, graft durability, repeat revascularization, and long-term survival. **Conclusions**: OPCAB should be considered an individualized strategy rather than a universal alternative to on-pump CABG. Optimal outcomes depend on patient selection, surgical expertise, vigilant monitoring, and timely correction of hemodynamic compromise. Potential neurological benefits should be interpreted in relation to the aortic-handling strategy, particularly when an anaortic/no-touch technique is used.

## 1. Introduction

Off-pump coronary artery bypass grafting is a surgical myocardial revascularization strategy in which coronary anastomoses are performed on the beating heart, without CPB. The technique was developed to avoid several physiological effects of extracorporeal circulation, including systemic inflammatory activation, coagulation disturbances, neurological complications, and end-organ dysfunction [1]. Contemporary evidence suggests that OPCAB achieves outcomes broadly comparable to those of ONCAB, particularly with respect to perioperative mortality and major adverse cardiovascular events, while avoidance of cardiopulmonary bypass may reduce selected complications, including perioperative stroke and transfusion requirements [2,3]. Nevertheless, OPCAB remains technically demanding, and concerns regarding incomplete revascularization, graft patency, repeat revascularization, and long-term outcomes continue to influence the choice between off-pump and on-pump surgery [4].

OPCAB is most often considered when avoidance of CPB or manipulation of the ascending aorta is expected to be beneficial. Potential candidates include selected patients with advanced aortic atherosclerosis, previous cerebrovascular disease, chronic kidney disease, chronic obstructive pulmonary disease, impaired left ventricular function, left main coronary artery disease, acute myocardial infarction, or previous cardiac surgery [5]. However, the potential reduction in perioperative stroke depends on the aortic-handling strategy, as OPCAB does not necessarily eliminate ascending aortic manipulation. An anaortic/no-touch technique, which avoids manipulation of the ascending aorta, may offer additional neurological protection in selected patients. The technique has also been incorporated into selected hybrid and combined procedures [6,7]. Conversely, cardiogenic shock, severe ischemia-related arrhythmias, marked hemodynamic instability, severe ventricular dysfunction, or coronary anatomy that makes safe exposure and complete revascularization difficult may limit the feasibility of OPCAB [8].

Successful OPCAB therefore depends on careful patient selection, detailed preoperative assessment, surgical expertise, and close coordination between the surgical and anesthesia teams. The anesthesiologist must anticipate rapid physiological changes caused by cardiac displacement, mechanical stabilization, and temporary coronary occlusion, particularly when the lateral or posterior coronary territories are exposed [9]. This narrative review summarizes the anesthetic, hemodynamic, and perioperative considerations that are most relevant to contemporary OPCAB practice and places these considerations in the context of current evidence comparing OPCAB with ONCAB.

## 2. Literature Search and Review Approach

This narrative review aimed to summarize and critically discuss the evidence on anesthetic, hemodynamic, and perioperative management in adults undergoing OPCAB. A literature search was conducted in PubMed/MEDLINE and Scopus, supplemented by Google Scholar and screening of reference lists from relevant systematic reviews, meta-analyses, guidelines, and landmark publications. The search covered publications from January 2000 through August 2026.

Medical Subject Headings (MeSH), where applicable, and free-text terms were combined using Boolean operators. The main search concepts included “off-pump coronary artery bypass,” OPCAB, “beating-heart coronary surgery,” “off-pump CABG,” anesthesia/anesthesia, perioperative management, hemodynamic monitoring, goal-directed therapy, transesophageal echocardiography, enhanced recovery, fast-track anesthesia, vasopressors, inotropes, hemodynamic optimization, and clinical outcomes. Search terms were adapted to the indexing and search functions of each database.

Studies were considered relevant if they addressed anesthetic management, hemodynamic monitoring or optimization, perioperative complications, or clinical outcomes related to OPCAB in adults. Priority was given to systematic reviews, meta-analyses, randomized controlled trials, observational studies, clinical guidelines, and publications providing important physiological or historical context. Conference abstracts, duplicate records, non-English publications, and studies without sufficient methodological information were excluded. Selected publications before January 2000 were retained when necessary to support foundational concepts, validated risk-assessment tools, and the historical development of perioperative cardiac risk evaluation.

The literature was selected according to relevance to the review objectives, study design, methodological limitations, consistency of findings, and clinical applicability. Attention was given to patient selection, surgical expertise, completeness of revascularization, conduit strategy, aortic manipulation, and follow-up duration when interpreting comparisons between OPCAB and ONCAB.

As this was a narrative review, no registered systematic-review protocol, formal risk-of-bias assessment, certainty-of-evidence grading, or quantitative synthesis was undertaken. The search and selection process supported a clinically focused discussion rather than an exhaustive systematic evidence assessment. The authors critically considered conflicting findings and the limitations of the available evidence when developing the clinical interpretation.

## 3. Anesthetic and Perioperative Considerations

The anesthetic management of OPCAB differs from conventional ONCAB because the heart remains beating throughout the operation and must be displaced and stabilized to expose the coronary targets. Cardiac manipulation and temporary coronary occlusion can produce rapid changes in preload, ventricular filling, myocardial perfusion, cardiac output, and rhythm. The principal anesthetic objective is therefore to preserve myocardial oxygen supply and systemic tissue perfusion while limiting increases in myocardial oxygen demand during periods of mechanical instability.

Successful management requires close communication between the anesthesiologist and surgeon, particularly during cardiac positioning and distal coronary anastomosis. Appropriate monitoring, anticipation of hemodynamic changes, and prompt treatment of hypotension, ischemia, ventricular dysfunction, and arrhythmias are central to completing OPCAB safely and avoiding unplanned conversion to CPB.

### 3.1. Preoperative Evaluation

Preoperative assessment should be individualized according to the severity of coronary artery disease, ventricular function, functional capacity, comorbidities, coronary anatomy, and the anticipated technical demands of OPCAB. The medical history and examination should identify conditions that may influence perioperative risk, including hypertension, diabetes, dyslipidemia, previous cerebrovascular disease, chronic kidney dysfunction, pulmonary disease, and previous cardiac surgery.

Formal cardiac-surgical risk estimation can be supported by EuroSCORE and EuroSCORE II, while functional status, ventricular function, coronary anatomy, comorbidities, and procedural complexity should be considered alongside any numerical risk estimate [10,11,12]. Risk assessment tools developed primarily for noncardiac surgery, such as the Revised Cardiac Risk Index, American College of Surgeons National Surgical Quality Improvement Program (ACS NSQIP) Surgical Risk Calculator, and National Surgical Quality Improvement Program–Myocardial Infarction or Cardiac Arrest (NSQIP-MICA), may provide general perioperative context but should not replace cardiac-surgical risk models when evaluating CABG candidates [13,14,15]. Functional capacity can be assessed with validated measures such as the Duke Activity Status Index [16,17]. In patients undergoing elevated-risk surgery, B-type natriuretic peptide (BNP) or N-terminal pro-B-type natriuretic peptide (NT-proBNP) and cardiac troponin may provide additional information in appropriate clinical settings [18].

Risk assessment should therefore not rely on a single score. Formal models should be interpreted together with functional status, left ventricular function, coronary anatomy, comorbidities, aortic pathology, and the anticipated technical complexity of the procedure. This integrated assessment is particularly important when deciding whether OPCAB is appropriate for an individual patient.

### 3.2. Monitoring and Anesthesia Techniques

Monitoring during OPCAB is particularly important because cardiac displacement, mechanical stabilization, and temporary coronary occlusion can rapidly alter ventricular filling, cardiac output, and myocardial perfusion. Standard monitoring includes continuous electrocardiography with ST-segment analysis, invasive arterial blood pressure, pulse oximetry, temperature, urine output, coagulation parameters, neuromuscular monitoring, and appropriate assessment of anesthetic depth. Pulmonary artery catheterization may provide additional information on cardiac output, pulmonary artery pressures, central venous pressure (CVP), and mixed venous oxygen saturation (SvO_2_) in selected patients, particularly those with severe ventricular dysfunction, pulmonary hypertension, or persistent hemodynamic instability [19,20]. Pressure measurements may be affected by cardiac displacement and altered geometry and should not be interpreted as direct measures of ventricular preload in isolation.

Transesophageal echocardiography (TEE) provides complementary information on ventricular function, regional wall motion, valvular function, ventricular interdependence, right ventricular compression, and left ventricular outflow tract obstruction. It is particularly useful when the cause of hemodynamic deterioration is unclear or electrocardiographic interpretation is difficult during cardiac displacement [21]. TEE findings can help distinguish mechanical impairment from myocardial ischemia and guide decisions regarding cardiac repositioning, fluid administration, vasoactive support, or conversion to CPB. However, image acquisition and interpretation may become more difficult during substantial cardiac displacement; TEE should therefore complement, rather than replace, continuous clinical and hemodynamic assessment.

General anesthesia remains the standard technique for OPCAB and may be maintained with volatile anesthetics or total intravenous anesthesia (TIVA). Volatile anesthetics have been associated with potential myocardial protective effects, whereas propofol-based TIVA provides predictable hypnosis and can be incorporated into fast-track pathways. Short-acting opioids and intermediate-duration neuromuscular blocking agents are commonly used to facilitate early emergence and extubation [22].

Enhanced Recovery After Cardiac Surgery (ERACS) principles increasingly inform perioperative management. These pathways emphasize multimodal, opioid-sparing analgesia, appropriate fluid management, early extubation, early mobilization, and reduction in intensive care and hospital length of stay [22]. Their implementation should be individualized according to patient condition and surgical complexity.

The available evidence does not establish the superiority of volatile anesthesia or TIVA in patients undergoing CABG. A systematic review of randomized studies involving on-pump and off-pump CABG reported better hemodynamic performance and a lower requirement for inotropic support with volatile anesthetics; however, these findings should be interpreted cautiously because of heterogeneity and methodological limitations [23]. An updated meta-analysis of 89 randomized trials involving more than 14,000 patients found no significant differences between volatile anesthesia and TIVA in operative or one-year mortality, myocardial infarction, stroke, heart failure, arrhythmias, acute kidney injury, or other major postoperative complications [24]. Similarly, a randomized study specifically involving OPCAB reported comparable postoperative recovery, opioid consumption, extubation time, and hospital length of stay with propofol-based TIVA and balanced volatile anesthesia [25]. Although differences in physiological performance may be clinically relevant, they do not establish superior clinical outcomes. Anesthetic technique should therefore be individualized according to patient characteristics, anticipated hemodynamic changes, institutional practice, and team experience.

Hemodynamic management should anticipate the effects of cardiac positioning. Preload may be supported with intravenous fluids or temporary Trendelenburg positioning, while vasopressors such as norepinephrine or vasopressin may be used to maintain adequate perfusion pressure. Selected patients may require inotropic support, including milrinone, to improve ventricular performance [26,27]. Heart rate should be controlled to limit myocardial oxygen consumption, and adjustments in positive end-expiratory pressure and tidal volume may help reduce right ventricular compression during cardiac displacement [28]. Goal-directed hemodynamic management and individualized vasoactive therapy may further support adequate tissue perfusion [28,29].

Hemodynamic targets should be interpreted in the context of baseline physiology and the stage of surgery. Maintaining a mean arterial pressure (MAP) above 70 mmHg and an SvO_2_ above 60% has been proposed as a practical approach to preserving systemic perfusion during OPCAB [28]. These values are not universally validated thresholds proven to improve clinical outcomes and should be individualized according to baseline blood pressure, ventricular function, coronary perfusion requirements, cardiac output, and other indicators of tissue perfusion.

Dynamic indices such as stroke volume variation (SVV) and pulse pressure variation (PPV) may assist in assessing fluid responsiveness, but their reliability is limited during OPCAB because cardiac displacement and mechanical stabilization alter cardiac geometry, ventricular filling, and loading conditions. Ventilation, cardiac rhythm, and intrathoracic pressure may further affect these measurements. SVV and PPV should therefore not be used in isolation to guide fluid administration. When fluid responsiveness is uncertain, their interpretation should be integrated with cardiac output, arterial pressure, TEE findings, and the response to carefully selected interventions. SvO_2_ provides an additional estimate of the balance between systemic oxygen delivery and consumption, while a rise in CVP relative to pulmonary artery diastolic pressure may suggest mechanical cardiac compression or impaired ventricular filling.

Regional analgesia may be incorporated into multimodal pain management, but high thoracic epidural analgesia and intrathecal morphine should not be considered routine techniques. Although high thoracic epidural analgesia may improve postoperative pain control and respiratory function, these benefits must be weighed against the risk of neuraxial hematoma, particularly in patients receiving antithrombotic therapy [30,31,32].

In CABG, aspirin is often continued and P2Y12 inhibitors may have been administered before surgery. Decisions about interrupting antiplatelet therapy should balance ischemic risk against surgical bleeding. Concomitant anticoagulants, thrombocytopenia, renal dysfunction, and traumatic needle placement may further increase bleeding risk. Although OPCAB avoids cardiopulmonary bypass, systemic intraoperative heparinization remains necessary. Managing an epidural catheter can therefore be challenging, particularly at removal. Current American Society of Regional Anesthesia and Pain Medicine (ASRA) guidance advises against maintaining neuraxial catheters during full anticoagulation for cardiac surgery. ASRA and European Society of Anaesthesiology and Intensive Care and European Society of Regional Anaesthesia and Pain Therapy (ESAIC/ESRA) guidelines emphasize appropriate timing of neuraxial procedures and catheter removal in relation to antithrombotic treatment [31,32].

Intrathecal morphine avoids the need for an indwelling epidural catheter but does not eliminate the bleeding risk associated with neuraxial needle placement. Its analgesic and opioid-sparing benefits must also be balanced against delayed respiratory depression, pruritus, nausea, and the need for appropriate postoperative monitoring. Patient selection should consider surgical urgency, antiplatelet exposure, planned heparinization and postoperative antithrombotic therapy, coagulation status, respiratory function, and available alternatives. High thoracic epidural analgesia combined with general anesthesia has also been investigated as an adjunct to cardiac surgery [30].

Neuraxial analgesia should therefore be considered only in carefully selected patients after assessing bleeding risk, antithrombotic treatment, respiratory status, and expected analgesic benefit. When these risks cannot be acceptably managed, multimodal systemic analgesia or an appropriate non-neuraxial regional technique may be preferable. Necessary antithrombotic treatment should not be compromised solely to facilitate neuraxial analgesia. The final approach should be agreed upon by the anesthesia and cardiac surgery teams.

Intraoperative care should also address ventilation, temperature control, coagulation, blood conservation, and analgesia. Lung-protective ventilation using appropriate tidal volumes and individualized positive end-expiratory pressure may help limit ventilator-associated lung injury and postoperative pulmonary complications [33]. Maintaining normothermia and applying evidence-based patient blood management strategies are important to preserve coagulation and reduce bleeding and transfusion requirements [34]. Multimodal, opioid-sparing analgesia, including regional techniques when appropriate, may improve pain control and support early extubation and recovery [22,35]. Parasternal blocks and surgical wound infiltration are additional options following median sternotomy and are recommended in the PROSPECT procedure-specific guidelines [35]. Evidence supporting these techniques in OPCAB remains limited, and the potential benefits should be considered alongside the basic systemic analgesics used in contemporary practice. In patients receiving antithrombotic therapy, the anatomical site, compressibility, and potential consequences of bleeding must be assessed in accordance with ASRA and ESAIC/ESRA guidance [31,32]. These measures should be individualized and integrated into the overall perioperative care pathway.

Preoperative patient blood management should include early identification of anemia, investigation of its underlying causes, and appropriate treatment before elective cardiac surgery. The 2024 EACTS/EACTAIC guidelines recommend investigating and treating newly diagnosed anemia and support consideration of iron supplementation in patients with iron-deficiency anemia. Oral iron may be considered when sufficient time is available, whereas intravenous iron may be appropriate when the time before surgery is limited. A randomized controlled trial specifically involving patients undergoing OPCAB demonstrated that preoperative intravenous ferric carboxymaltose reduced red blood cell transfusion requirements and improved hematological parameters compared with placebo [34,36].

Pulmonary protection remains important because avoiding cardiopulmonary bypass does not eliminate postoperative pulmonary complications. Ventilation should be individualized using lung-protective tidal volumes, appropriate positive end-expiratory pressure (PEEP), and avoidance of excessive airway pressures. Settings should be reassessed during cardiac displacement because changes in intrathoracic pressure and cardiac geometry may affect venous return and right ventricular function. Adequate oxygenation, avoidance of unnecessary fluid overload, effective analgesia, and timely extubation in appropriately selected patients may further support respiratory recovery, particularly in patients with pre-existing pulmonary disease [33].

Renal protection is particularly important in patients with chronic kidney disease, diabetes mellitus, or other risk factors for postoperative acute kidney injury (AKI). Risk is multifactorial and may be increased by inadequate renal perfusion, impaired oxygen delivery, hemodynamic instability, and nephrotoxic exposures. Management should include individualized fluid and vasoactive therapy, monitoring of urine output and serum creatinine, avoidance of avoidable nephrotoxic exposures, and appropriate glycemic control. In patients at high risk of AKI, a structured kidney-protection approach based on established cardiac-surgery recommendations may support early detection and prevention of further renal injury [37].

### 3.3. Intraoperative Hemodynamic Changes

Hemodynamic instability during OPCAB is primarily related to the mechanical manipulation required to expose coronary arteries while the heart continues to beat. Elevation and rotation of the heart for lateral and posterior targets can increase right- and left-sided filling pressures, reduce ventricular end-diastolic volumes and cardiac output, and decrease mixed venous oxygen saturation [19,38]. The magnitude of these changes depends on the coronary territory being grafted and is generally greatest during circumflex artery revascularization, when cardiac displacement is most pronounced [39,40].

Mechanical stabilization can further impair ventricular filling and regional contractility. Local pressure applied to the myocardium may reduce global stroke volume, while displacement may compress the right ventricle or right ventricular outflow tract and reduce venous return to the left ventricle [41,42]. Cardiac displacement may also alter mitral valve geometry and exacerbate mitral regurgitation, particularly during positioning for coronary anastomosis. Intraoperative TEE enables assessment of positional changes in mitral regurgitation and their associated hemodynamic consequences [43].

Temporary interruption of coronary blood flow during distal anastomosis is another potential cause of myocardial ischemia and hemodynamic deterioration. Coronary occlusion may produce ST-segment changes, new regional wall-motion abnormalities, impaired ventricular function, and arrhythmias. The clinical response depends on the severity and location of the coronary lesion, collateral circulation, and the myocardial territory at risk. Manipulation or occlusion of the right coronary artery is particularly important because impaired conduction-system perfusion may cause bradyarrhythmia or complete heart block [44,45].

Recognition of the underlying mechanism requires consideration of the timing of deterioration in relation to cardiac displacement, mechanical stabilization, and temporary coronary occlusion, together with arterial pressure, heart rate and rhythm, ECG findings, cardiac output when available, and TEE findings when appropriate. Impaired ventricular filling or reduced venous return may indicate displacement-related mechanical effects, whereas new ischemic changes or regional wall-motion abnormalities raise concern for myocardial ischemia. Worsening ventricular function or new arrhythmias may indicate additional myocardial or conduction-system impairment [21,39,40,41,42,43,44,45].

Treatment should target the suspected cause rather than rely on a uniform set of interventions. When reduced preload is suspected without significant mechanical compression or congestion, cautious fluid administration may be considered. Trendelenburg positioning may improve venous return in selected patients, but its effect should be reassessed. Vasopressors may be appropriate when inadequate vascular tone contributes to hypotension, whereas inotropic support may be considered when impaired myocardial contractility contributes to low cardiac output. Bradyarrhythmias and other rhythm disturbances should be managed according to their type and hemodynamic consequences. Ventilator settings may require adjustment when intrathoracic pressure or right ventricular loading contributes to instability. If deterioration develops during cardiac displacement or mechanical stabilization, the team should promptly reassess cardiac position and stabilizer pressure; reducing or releasing displacement may be necessary when mechanical impairment is suspected.

Following each intervention, arterial pressure, heart rate and rhythm, ECG changes, cardiac output when available, and evidence of ventricular function and systemic perfusion should be reassessed. TEE can provide additional information about ventricular filling, compression, and regional wall motion, although findings must be interpreted in the context of operative position and other monitoring data. Failure to improve, recurrent deterioration, or ongoing myocardial ischemia should prompt immediate reconsideration of the underlying mechanism and the safety of continuing the anastomosis.

A reduction in cardiac output does not invariably indicate inadequate tissue perfusion if systemic oxygen delivery remains sufficient. This interpretation requires caution in the presence of active myocardial ischemia or rapidly worsening hemodynamics. Cardiac output should therefore be interpreted alongside arterial pressure, SvO_2_, lactate, urine output, ECG findings, and echocardiographic assessment, as no single parameter reliably excludes inadequate perfusion. MAP and SvO_2_ should not be regarded as universal thresholds or substitutes for clinical assessment. Persistent hypotension, ongoing ischemia, severe ventricular dysfunction, refractory arrhythmias, or failure to respond promptly to corrective measures should prompt early consideration of releasing cardiac displacement and converting to CPB rather than continuing an unsafe operative position [28,39,40,41,42,43,44,45].

**Figure 1:**
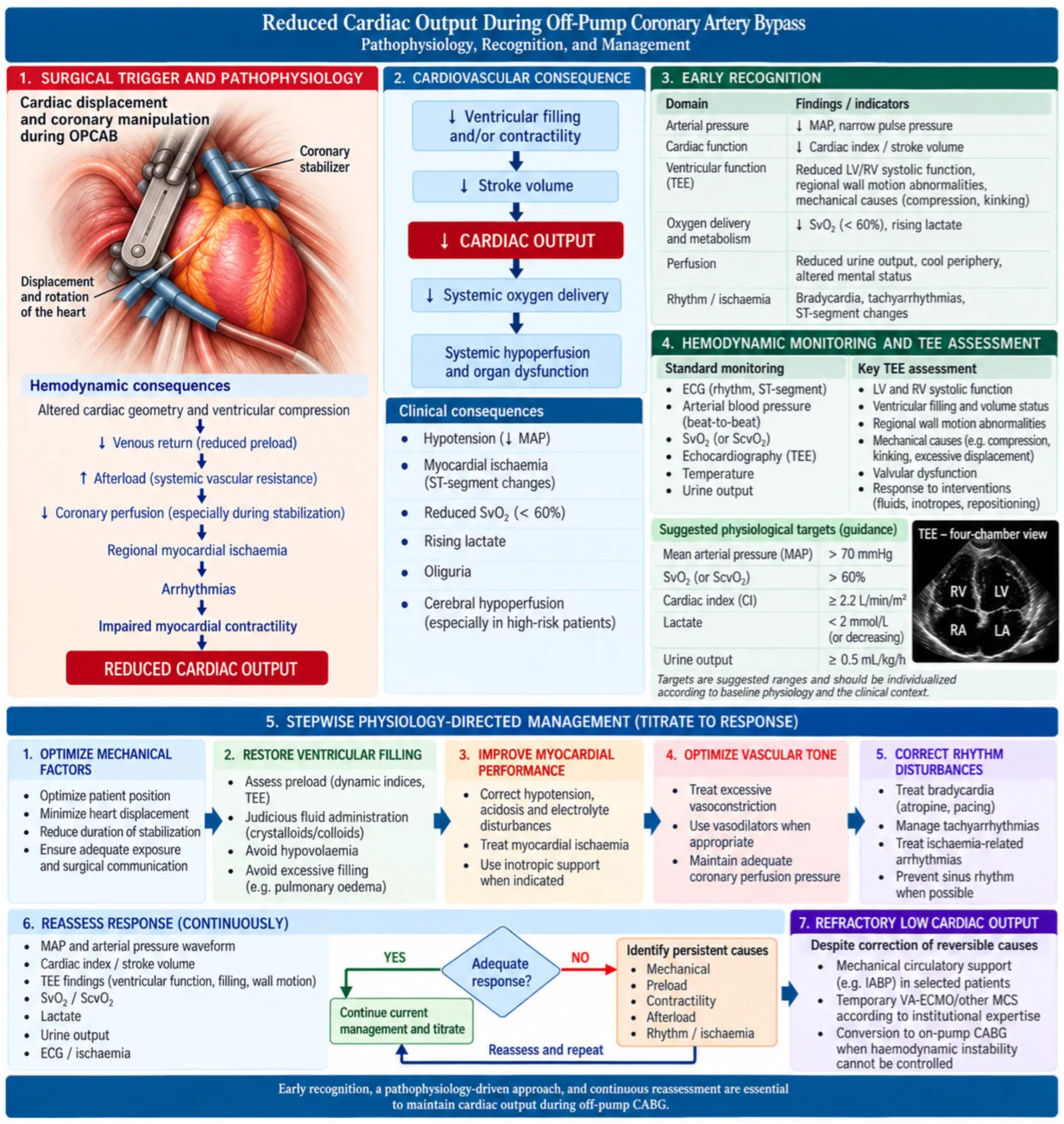
Proposed illustrative framework for the recognition, monitoring, and physiology-guided management of reduced cardiac output during off-pump coronary artery bypass grafting (OPCAB). TEE, transesophageal echocardiography; ECG, electrocardiography; MAP, mean arterial pressure; LV, left ventricle; RV, right ventricle; SV, stroke volume; SVR, systemic vascular resistance; ScvO_2_, central venous oxygen saturation; SvO_2_, mixed venous oxygen saturation; CI, cardiac index; ABG, arterial blood gas; MCS, mechanical circulatory support; VA-ECMO, venoarterial extracorporeal membrane oxygenation; and CPB, cardiopulmonary bypass. Physiological targets are provided as suggested guidance rather than universal treatment thresholds. Management should be individualized according to the clinical context and response to intervention.

As illustrated in Figure 1, hemodynamic deterioration during OPCAB may reflect the combined effects of altered cardiac geometry, impaired ventricular filling, increased afterload, myocardial ischemia, and rhythm disturbances. Early recognition, mechanism-directed intervention, and continuous reassessment are essential to restore hemodynamic stability and determine when continued off-pump surgery is no longer safe.

Protocolized hemodynamic management may improve intraoperative stability during OPCAB. In a small, nonrandomized comparison, Khanh et al. reported that an early goal-directed therapy protocol was associated with lower lactate concentrations, reduced vasoactive and inotropic requirements, and shorter durations of mechanical ventilation, ICU stay, and hospital stay compared with conventional management [46]. Their approach integrated stroke volume variation (SVV), systemic vascular resistance (SVR), hemoglobin concentration, and cardiac index (CI) to guide fluid administration, vasopressor therapy, transfusion, and inotropic support. However, these findings are hypothesis-generating and do not establish the effectiveness of protocolized management during OPCAB. The original algorithm used a hemoglobin concentration below 10 g/dL as a transfusion trigger and predefined SVV, SVR, and CI thresholds to guide treatment [46]. These thresholds reflect the study protocol and should not be considered universal treatment targets. The variables used in the protocol must be interpreted in the context of cardiac displacement, ventricular function, and the limitations of dynamic indices discussed in Section 3.2.

The 2024 EACTS/EACTAIC guidelines on patient blood management in adult cardiac surgery support a restrictive red blood cell transfusion strategy, with a threshold of ≤7.5 g/dL supported by the guideline recommendations [34]. Transfusion decisions during OPCAB should consider hemoglobin concentration alongside active bleeding, evidence of myocardial ischemia, hemodynamic status, and systemic oxygen delivery. Fluid administration and vasoactive or inotropic support should likewise be individualized according to the underlying mechanism and response to treatment. Persistent severe instability, ongoing myocardial ischemia, refractory arrhythmias, or failure to respond promptly to corrective measures should prompt early consideration of conversion to ONCAB rather than continued reliance on predefined numerical thresholds.

### 3.4. Practical Anesthetic Management During Off-Pump Coronary Artery Bypass Grafting

#### 3.4.1. Induction and Initial Hemodynamic Stabilization

Anesthetic induction in patients with critical coronary artery disease requires careful prevention of hypotension, tachycardia, and increased myocardial oxygen demand. The choice and doses of induction agents and opioids should be individualized according to ventricular function, hemodynamic status, and the severity of coronary disease. Invasive arterial pressure monitoring should be established before induction whenever feasible, with vasoactive drugs readily available. The main objectives are to maintain coronary perfusion pressure, avoid significant changes in heart rate, and promptly identify and manage instability [8,28].

#### 3.4.2. Preparation Before Cardiac Displacement

Before cardiac displacement, the anesthesiologist and surgeon should confirm hemodynamic stability, adequate oxygenation and ventilation, appropriate vascular access, and the availability of vasoactive support. Baseline hemodynamic parameters should be recorded, and TEE should be available when appropriate to assess ventricular function and detect new regional wall-motion abnormalities during cardiac manipulation [21,28]. The team should agree in advance on when to pause the procedure, release cardiac displacement, or proceed to CPB if instability cannot be promptly corrected [28].

#### 3.4.3. Recognition and Management of Intraoperative Hemodynamic Deterioration

Continuous electrocardiographic monitoring, including ST-segment analysis, should be combined with assessment of arterial pressure, heart rate, cardiac output when available, and TEE findings. New ischemic changes, regional wall-motion abnormalities, hypotension, arrhythmias, or worsening ventricular function require immediate communication between the anesthesiologist and surgeon. Management should follow the mechanism-directed approach described in Section 3.3 and may include reducing cardiac displacement, reassessing coronary occlusion or mechanical stabilization, optimizing preload and vascular tone, and administering vasoactive or inotropic support when indicated. Persistent ischemia or hemodynamic instability despite corrective measures should prompt reassessment of whether it is safe to continue the anastomosis [21,28,39,40,41,42,43,44,45].

#### 3.4.4. Preparation for Urgent Conversion to On-Pump Surgery

Conversion to CPB should be anticipated rather than treated as an unexpected last resort. Before surgery, the team should establish conversion criteria, individual responsibilities, the availability of the perfusion team and required equipment, and clear communication pathways. Persistent hypotension, refractory ischemia, severe ventricular dysfunction, malignant arrhythmias, or hemodynamic collapse despite corrective measures should prompt early consideration of conversion. Although emergency conversion carries substantial risk, timely recognition and decision-making are essential to prevent prolonged myocardial and systemic hypoperfusion [8,28].

## 4. OPCAB Versus ONCAB: Current Evidence and Clinical Implications

The relative benefits of OPCAB and ONCAB depend on patient characteristics, surgical expertise, conduit selection, completeness of revascularization, and the extent of aortic manipulation. Although OPCAB may reduce selected perioperative complications, these potential benefits must be balanced against the risks of incomplete revascularization and repeat intervention. Evidence varies in study design and follow-up, so early postoperative findings should not automatically be interpreted as evidence of long-term benefit. Table 1 summarizes the main findings alongside the reported follow-up periods and key limitations, distinguishing early perioperative outcomes from longer-term results.

In our view, the clinical value of OPCAB should not be judged solely by the avoidance of cardiopulmonary bypass or by differences in individual postoperative outcomes. Its potential benefits should be considered alongside the physiological challenges associated with cardiac manipulation, the ability to achieve complete revascularization, and the experience of both the surgical and anesthetic teams. Patients at increased risk of complications related to cardiopulmonary bypass or aortic manipulation may benefit from OPCAB, provided that adequate myocardial and systemic perfusion can be maintained and complete revascularization remains achievable. Conversely, when cardiac displacement causes persistent instability or compromises surgical exposure, continuing an off-pump approach may outweigh its anticipated benefits. We therefore consider patient selection, coordinated intraoperative decision-making, and timely conversion when necessary to be more clinically relevant than treating OPCAB and ONCAB as universally competing techniques.

The economic comparison between OPCAB and ONCAB remains context-dependent. Potential savings from reduced perioperative resource use may be offset by the costs of specialized equipment, intraoperative conversion, or subsequent revascularization. A cost-effectiveness analysis alongside the ROOBY trial found higher 1-year costs with OPCAB when late conversion to on-pump surgery was included; however, the difference was no longer statistically significant when these conversions were excluded. These findings highlight the importance of surgical strategy and local healthcare costs when evaluating the economic implications of each approach [47]. Overall, the available evidence does not establish a consistent cost advantage for either OPCAB or ONCAB. Economic outcomes vary according to surgical strategy, conversion rates, resource utilization, and institutional costs [48].

From an anesthetic perspective, successful OPCAB depends not only on maintaining hemodynamic stability but also on recognizing the underlying mechanisms of deterioration during cardiac manipulation. The ability to distinguish impaired ventricular filling, mechanical compression, myocardial ischemia and ventricular dysfunction is essential for selecting appropriate interventions. Continuous communication between the anesthesiologist and surgeon, repeated assessment of the response to treatment, and early consideration of conversion to cardiopulmonary bypass when instability persists are central to patient safety. These considerations highlight the importance of a coordinated multidisciplinary approach rather than reliance on predefined hemodynamic targets alone.

**Table 1:** Recent evidence comparing off-pump and on-pump coronary artery bypass grafting.

Study	Design/Population	Principal Findings	Clinical Interpretation	Follow-Up Period
Pansani et al. [49]	Systematic review and meta-analysis; left main coronary artery disease; 18 studies (1 RCT and 17 observational studies)	Short-term pooled results favored OPCAB for mortality and renal complications; stroke and MI were not significantly different. Fewer grafts were performed with OPCAB.	May support OPCAB in selected patients, but the evidence is predominantly observational and does not establish superiority. The lower graft number is an important consideration when judging completeness of revascularization.	Primarily in-hospital/30-day outcomes; four studies contributed mid-term data (mean follow-up 38.1 months), with no significant mortality difference in that subset.
Comanici et al. [50]	Systematic review and meta-analysis/meta-regression; 7 studies, 5417 patients; total arterial grafting in both groups	Overall perioperative complications did not differ significantly. TAG-OPCAB was associated with lower short-term mortality (OR 0.48, 95% CI 0.26–0.89), less low cardiac output, AF and MACCEs, and shorter intubation duration and hospital stay.	Favorable short-term outcomes are promising, but nonrandomized treatment allocation, patient selection, surgical expertise, and differences in graft configurations limit causal attribution. Long-term benefit remains unproven.	Predominantly perioperative/short-term; only limited longer-term data, insufficient to establish long-term outcomes.
Rai et al. [51]	Retrospective cohort study; 108 patients	Comparable early mortality and postoperative complications; less postoperative inotropic support reported with OPCAB.	Hypothesis-generating only; small sample size and non-randomized treatment selection limit causal conclusions.	In-hospital and 30-day outcomes.
He et al. [52]	Meta-analysis of 28 randomized trials; 16,090 patients	Lower perioperative stroke risk with OPCAB, but higher repeat revascularization and less favorable long-term outcomes were reported.	Potential early neurological benefit must be balanced against reintervention and longer-term outcomes; differences in expertise, crossover, grafting completeness and conduit choice matter.	Reported follow-up ranged from 1 month to 10 years; some trials reported only in-hospital outcomes.
Cappellaro et al. [53]	Systematic review and meta-analysis; 6 studies, 1687 patients with COPD	Shorter mechanical ventilation time with OPCAB; no significant differences in mortality or several major postoperative complications.	May indicate an early respiratory advantage in some patients with COPD but does not establish an overall clinical benefit for this subgroup.	Follow-up varied across studies; evidence mainly concerns perioperative/early postoperative outcomes, with no single uniform follow-up period reported.
Xu et al. [54]	Retrospective cohort study; 1269 patients with diabetes	Lower postoperative stroke and AF and lower 5-year mortality associated with OPCAB; 5-year MI and stroke did not differ significantly.	Observational associations do not show that diabetes itself predicts greater benefit from OPCAB; residual confounding and selection effects remain possible.	In-hospital, 2-year and 5-year outcomes.
Comanici et al. [55]	Updated systematic review, meta-analysis and meta-regression; 22 studies, 69,449 patients (3 RCTs; majority observational)	No significant difference in 10-year all-cause mortality (HR 1.00, 95% CI 0.92–1.08).	Does not support a general long-term survival advantage for either strategy; interpretation is limited by heterogeneity and the predominance of observational evidence.	10-year all-cause mortality endpoint.

Abbreviations: AF, atrial fibrillation; COPD, chronic obstructive pulmonary disease; CPB, cardiopulmonary bypass; ICU, intensive care unit; MACCE, major adverse cardiovascular and cerebrovascular events; MI, myocardial infarction; ONCAB, on-pump coronary artery bypass grafting; OPCAB, off-pump coronary artery bypass grafting; RCT, randomized controlled trial; TAG, total arterial grafting; TAG-ONCAB, total arterial grafting with on-pump CABG; TAG-OPCAB, total arterial grafting with off-pump CABG.

### 4.1. Evidence in Selected Patient Populations

In patients with left main coronary artery disease, a systematic review and meta-analysis found that OPCAB was associated with lower short-term mortality and fewer renal complications; stroke and myocardial infarction rates were not significantly different from ONCAB [49]. However, the analysis included only one randomized trial, and fewer grafts were performed in the OPCAB group. Mortality did not differ significantly between OPCAB and ONCAB over a mean follow-up of 38.1 months. These findings support OPCAB as an option for selected patients but do not establish its superiority over ONCAB, particularly with respect to long-term outcomes.

The comparison between OPCAB and ONCAB may also be influenced by the revascularization strategy. A meta-analysis of seven studies involving 5417 patients specifically compared total arterial grafting performed with and without cardiopulmonary bypass (TAG-OPCAB vs. TAG-ONCAB) [50]. Overall perioperative complications did not differ significantly, while TAG-OPCAB was associated with lower short-term mortality (OR 0.48, 95% CI 0.26–0.89), less postoperative low cardiac output, atrial fibrillation and Major Adverse Cardiac and Cerebrovascular Events (MACCEs), and shorter intubation and hospital stay. These findings are limited to short-term outcomes, while evidence of long-term benefit remains insufficient. Moreover, the observed associations should not be interpreted as proof of a causal benefit of the off-pump technique, given the potential influence of patient selection, surgical expertise, and other confounding factors.

### 4.2. Early and Long-Term Outcomes

A small retrospective cohort of 108 patients reported comparable early mortality and postoperative complications with OPCAB and ONCAB, although use of postoperative inotropic support was less frequent with OPCAB [51]. The limited sample size and observational design mean this finding should not be taken as proof of a consistent hemodynamic advantage.

Longer-term comparisons are less consistent. A meta-analysis of 28 randomized trials involving 16,090 patients found a lower perioperative stroke risk with OPCAB but also reported higher repeat revascularization and less favorable long-term outcomes [52]. Follow-up ranged from one month to ten years, and some studies reported only in-hospital outcomes. Differences in surgeon experience, crossover between techniques, completeness of revascularization, conduit choice, and aortic manipulation may contribute to variation in results. In patients with chronic obstructive pulmonary disease, a systematic review found shorter mechanical ventilation time with OPCAB, but no significant differences in mortality or several other major complications [53]. Follow-up periods varied across the included studies, so this evidence mainly supports a possible early respiratory advantage rather than an overall clinical benefit. A retrospective cohort of 1269 patients with diabetes reported lower postoperative stroke and atrial fibrillation rates and lower five-year mortality with OPCAB; five-year myocardial infarction and stroke did not differ significantly [54]. As this was an observational association, residual confounding and treatment selection may explain some of the findings; current evidence does not support diabetes alone as an indication for OPCAB.

### 4.3. Overall Clinical Interpretation

An updated systematic review and meta-analysis of 22 studies involving 69,449 patients found no statistically significant difference in 10-year all-cause mortality between OPCAB and ONCAB (HR 1.00, 95% CI 0.92–1.08) [55]. Although the pooled estimate showed no statistically significant difference, substantial between-study heterogeneity and the predominance of observational evidence warrant cautious interpretation. Importantly, the absence of a statistically significant difference does not establish equivalence or non-inferiority between the two techniques.

Overall, the evidence does not support a universal preference for OPCAB or ONCAB. OPCAB may be advantageous in selected patients, particularly when avoidance of CPB or aortic manipulation is important; its benefits must be balanced against the possibility of incomplete revascularization, repeat intervention, reduced graft durability, and less favorable long-term outcomes. The decision should therefore be individualized according to coronary anatomy, comorbidities, aortic pathology, ventricular function, anticipated technical difficulty, and the experience of the surgical team. Importantly, avoiding CPB during OPCAB does not necessarily eliminate ascending aortic manipulation, as partial aortic clamping may still be required for proximal graft anastomoses. Therefore, any potential reduction in perioperative stroke risk should be interpreted in relation to the extent of aortic manipulation, particularly when an aortic or no-touch technique is employed, rather than attributed solely to the avoidance of CPB [56].

### 4.4. Surgical Expertise, Learning Curve, and Volume–Outcome Relationship

OPCAB is technically demanding, and its outcomes may depend partly on the experience of the surgeon and surgical team. The learning curve extends beyond performing coronary anastomoses on a beating heart to include patient selection, cardiac positioning and stabilization, maintenance of hemodynamic stability, achievement of complete revascularization, and timely recognition of the need for conversion to cardiopulmonary bypass. Limited experience during the adoption of OPCAB may therefore affect procedural performance and postoperative outcomes [57].

Studies investigating the relationship between surgeon or hospital volume and OPCAB outcomes have reported mixed findings. Sepehripour and Athanasiou identified a possible volume–outcome relationship but emphasized the limitations and inconsistency of the available evidence [57]. The European Multicenter Study on Coronary Artery Bypass Grafting (E-CABG) Registry also examined the influence of surgeon experience and center volume on OPCAB outcomes [58]. However, current evidence does not prove a universal minimum-volume threshold, and surgical volume alone cannot fully represent technical proficiency, team coordination, case complexity, or institutional resources.

Differences in surgical experience may also contribute to heterogeneity in comparisons between OPCAB and ONCAB across randomized trials, observational studies, and meta-analyses. The variation in operator proficiency, patient selection, conversion practices, conduit choice, and completeness of revascularization should be considered when interpreting pooled results, where relevant data are available. Clinically, choosing between OPCAB and ONCAB should reflect the individual patient’s characteristics, the anticipated benefits and risks of each approach, and the surgical team’s experience and available resources. This perspective recognizes surgical expertise as an important determinant of safe and effective revascularization, while acknowledging that experience alone cannot explain differences in outcomes or establish the superiority of either technique.

### 4.5. Limitations and Future Directions

This review has several limitations. First, it is a narrative rather than a systematic review; the search strategy and selection priorities were defined in advance, but the study selection and interpretation may still be influenced by authors’ judgment. Second, no formal risk-of-bias assessment, certainty-of-evidence grading, or quantitative synthesis were performed. Finally, heterogeneity in patient selection, surgical expertise, monitoring strategies, conversion thresholds, and outcome definitions limits direct comparison across studies. These limitations reinforce the need of cautious interpretation of comparative outcomes and for prospective studies evaluating standardized perioperative strategies in well-defined OPCAB populations.

## 5. Conclusions

OPCAB is an established revascularization strategy that can be valuable in carefully selected patients, particularly when an anaortic/no-touch technique is used. From an anesthetic perspective, the principal challenge is the rapid and sometimes profound hemodynamic disturbance produced by cardiac displacement, mechanical stabilization, and temporary coronary occlusion. Continuous monitoring, TEE when indicated, individualized preload and vasoactive management, and close surgical–anesthetic communication are therefore central to safe practice.

Current evidence does not demonstrate consistent clinical superiority of volatile anesthesia or TIVA. Comparative clinical studies suggest potential reductions in perioperative stroke, transfusion, renal complications, and hospital stay with OPCAB, but these possible early benefits must be balanced against concerns regarding complete revascularization, graft durability, repeat revascularization, and long-term survival. The potential reduction in perioperative stroke depends on the aortic-handling strategy, as OPCAB does not necessarily eliminate ascending aortic manipulation. An anaortic/no-touch technique, which avoids manipulation of the ascending aorta, may offer additional neurological protection in selected patients. OPCAB should be viewed as an individualized surgical strategy whose success depends on appropriate patient selection, experienced teams, and prompt recognition and treatment of hemodynamic compromise.

## Data Availability

No new data were created or analyzed in this study. Data sharing is not applicable to this narrative review. All information discussed in the manuscript is derived from previously published studies, which are cited in the reference list.
